# Glutamate Excitotoxicity Inflicts Paranodal Myelin Splitting and Retraction

**DOI:** 10.1371/journal.pone.0006705

**Published:** 2009-08-20

**Authors:** Yan Fu, Wenjing Sun, Yunzhou Shi, Riyi Shi, Ji-Xin Cheng

**Affiliations:** 1 Weldon School of Biomedical Engineering, Purdue University, West Lafayette, Indiana, United States of America; 2 Department of Basic Medical Sciences and Center for Paralysis Research, Purdue University, West Lafayette, Indiana, United States of America; 3 Department of Chemistry, Purdue University, West Lafayette, Indiana, United States of America; National Institutes of Health, United States of America

## Abstract

Paranodal myelin damage is observed in white matter injury. However the culprit for such damage remains unknown. By coherent anti-Stokes Raman scattering imaging of myelin sheath in fresh tissues with sub-micron resolution, we observed significant paranodal myelin splitting and retraction following glutamate application both *ex vivo* and *in vivo*. Multimodal multiphoton imaging further showed that glutamate application broke axo-glial junctions and exposed juxtaparanodal K^+^ channels, resulting in axonal conduction deficit that was demonstrated by compound action potential measurements. The use of 4-aminopyridine, a broad-spectrum K^+^ channel blocker, effectively recovered both the amplitude and width of compound action potentials. Using CARS imaging as a quantitative readout of nodal length to diameter ratio, the same kind of paranodal myelin retraction was observed with applications of Ca^2+^ ionophore A23187. Moreover, exclusion of Ca^2+^ from the medium or application of calpain inhibitor abolished paranodal myelin retraction during glutamate exposure. Examinations of glutamate receptor agonists and antagonists further showed that the paranodal myelin damage was mediated by NMDA and kainate receptors. These results suggest that an increased level of glutamate in diseased white matter could impair paranodal myelin through receptor-mediated Ca^2+^ overloading and subsequent calpain activation.

## Introduction

White matter of the central nervous system (CNS) is enriched in myelinated axons which are critical for reliable and efficient action potential conduction. The action potential is generated at nodes of Ranvier and propagates via saltatory conduction. Adjacent to the nodes are paranodes where axolemma and the lateral borders of myelin sheath are connected through the adhesion junctions. The integrity of paranodal domains is vital to fast action potential conduction along myelinated axons. Irreversible injury to white matter [1] leads to severe functional loss of the CNS in neurological disorders including stroke [2], multiple sclerosis (MS) [3], and spinal cord trauma [4]. Early electron microscopy studies showed submyelinic vacuoles and detachment of perinodal oligodendrocyte-axon loops in anoxic injury of rat optic nerves [5], [6]. Howell *et al*. observed the disruption of adhesion junctions within and adjacent to actively demyelinating white matter lesions in MS tissues [7]. Nevertheless, the culprit responsible for such paranodal myelin injury remains elusive to date.

Studies using various animal models have suggested glutamate excitotoxicity as a common pathway in white matter injury [8], [9]. Abnormally high glutamate levels were found in the cerebrospinal fluid of MS [3] and spinal cord injury [10] patients. Oligodendrocytes are known to be vulnerable to glutamate excitotoxicity through activation of glutamate receptors [11], [12]. Studies of *ex vivo* spinal dorsal column [13] and cerebral white matter [14] have shown that glutamate excitotoxicity could lead to death of oligodendrocytes and loss of axonal conduction. Nevertheless, whether and how glutamate impairs paranodal myelin remains to be investigated.

The current paper employs coherent anti-Stokes Raman scattering (CARS) microscopy to investigate glutamate-induced myelin damage in a spinal cord *ex vivo* and *in vivo*. CARS microscopy uses two pulsed lasers to coherently drive a specific molecular vibration by tuning the beating frequency, i.e., the frequency difference between the two lasers, to a Raman band [15]. It allows vibrational imaging at a speed that is 10^6^ times faster than spontaneous Raman microscopy. As a spiral membrane containing about 70% lipid by weight [16], CNS myelin produces a large CARS signal from the high-density CH_2_ groups. With a lateral resolution of 0.28 µm and an axial resolution of 0.70 µm, our CARS microscope could visualize paranodal myelin flanking a node of Ranvier [17]. Moreover, laser-scanning CARS microscopy has allowed label-free monitoring of lysophosphatidylcholine-induced myelin degradation [18]. In this work, we utilize the label-free and real-time imaging capability of CARS microscopy to quantitatively measure the paranodal myelin damage over a large number of axons. We show for the first time that glutamate toxicity causes splitting and retraction of paranodal myelin and consequent exposure of juxtaparanodal K^+^ channels. We further show that such paranodal structural changes result from glutamate receptor-mediated Ca^2+^ influx and subsequent activation of calpain.

## Materials and Methods

### Preparation of ex vivo spinal cord white matter

All animals used in this study were handled in strict accordance with NIH guidelines for the *Care and Use of Laboratory Animals*. The experimental protocol was approved by the Purdue Animal Care and Use Committee. Adult female guinea pigs of 350–500 g bodyweight and ages between 4–8 weeks old were anesthetized deeply with 80 mg/kg ketamine, 0.8 mg/kg acepromazine, and 12 mg/kg xylazine and perfused transcardially with cold Krebs' solution (NaCl 124 mM, KCl 2 mM, KH_2_PO_4_ 1.2 mM, MgSO_4_ 1.3 mM, CaCl_2_ 2 mM, dextrose 10 mM, NaHCO_3_ 26 mM, and sodium ascorbate 10 mM). The spinal cord was quickly removed from the vertebral column and placed in cold Krebs' solution bubbled with 95% O_2_/5% CO_2_. The extracted spinal cord was first split into two halves by mid-line sagittal division and then cut radially to separate the ventral white matter as previously described [19]. Spinal tissues were maintained in continuously oxygenated Krebs' solution for at least an hour at room temperature before imaging or recording.

### In vivo glutamate treatment

 7 Long-Evans Rats were anesthetized deeply with a mixture of 80 mg/kg ketamine and 5 mg/kg xylazine. A 3 mm×3 mm window on the spinal cord was exposed at T9-T11 by aseptic laminectomy. 3 mL sterile saline was subcutaneously injected into the rat during surgery. In the glutamate group (Glut) including 4 rats, 0.1 mL of 1 mM glutamate solution was gently dropped onto the exposed spinal cord. After 1 min, the glutamate solution was aspirated by gauge pad, and then another 0.1 mL glutamate solution was dropped. This process was repeated till the total volume of glutamate solution used was 1 mL. The last drop of glutamate solution was kept on the exposed spinal cord and the opened tissue and skin were stitched together. 12 h after the surgery, the rat was euthanized through transcardial perfusion with cold PBS (pH = 7.4), followed by PBS solution containing 4% paraformaldehyde. The spinal cord was extracted and post-fixed in 4% paraformaldehyde solution for at least 24 h. The exposed part and the upper part (toward the cervical part) were sectioned into 100 µm longitudinal sections by vibratome. 3 or 4 dorsal sections near the dorsal surface were imaged by CARS microscopy, and ratios of nodal length to nodal diameter were analyzed. 3 rats in the control group (Ctrl) were treated in the same way using 1 mL sterile saline.

### CARS and two-photon excited fluorescence (TPEF) imaging

 The CARS imaging system is shown in Supplementary Figure S1. The two beams at frequency ω_p_ and ω_s_ were generated from two tightly synchronized Ti:sapphire lasers (Mira 900/Sync-lock, Coherent Inc.). Both lasers have a pulse duration of 2.5 ps. The two beams were parallel-polarized and collinearly combined. A Pockels' cell was used to reduce the repetition rate from 78 MHz to 7.8 MHz. The overlapping beams were directed into a laser scanning microscope (FV300/IX70, Olympus Inc.) and focused into a sample through a 60X water immersion objective lens (numerical aperture = 1.2). The epi-detected CARS signal was collected in the backward direction with the same objective lens. The frequency difference between the pump and Stokes beams, ω_p_−ω_s_, was tuned to the symmetric CH_2_ vibration at 2840 cm^−1^. The same picosecond laser beams were also used for TPEF imaging of calcium indicators. The epi-detected TPEF signal was spectrally separated from the CARS signal by a dichroic splitter. Both CARS and TPEF signals were detected with the same type of photomultiplier tube (PMT, R3896, Hamamatsu, Japan). The average pump and Stokes laser power at the sample were around 3.6 mw and 1.2 mw, respectively. No photodamage to myelin was observed. For real-time CARS imaging, the isolated ventral white matter strip about 1 cm in length was directly mounted on a glass-bottom culture dish (#P35G-1.5-14-C, MatTek Co. Ashland, MA) and kept in oxygen-bubbled Krebs' solution. For Ca^2+^ imaging, the cords were first incubated in a Ca^2+^-free Krebs' solution (Ca^2+^ was replaced with Mg^2+^) that contained 40 µM Oregon Green 488 AM (BAPTA-2, Molecular Probes, Eugene, OR) for 2 h and then washed with normal Krebs' solution (containing 2 mM Ca^2+^) prior to imaging. Our CARS imaging system uses a water immersion objective. Since accelerated water evaporation at 37°C would therefore make long-time imaging difficult, all the imaging experiments were carried out at room temperature (23°C).

### Electrophysiological recording

 Compound action potential (CAP) measurements in an *ex vivo* spinal cord ventral column were carried out by using a double sucrose gap chamber [19], as shown in Supplementary Figure S2. A strip of spinal cord ventral white matter approximately 40 mm in length was placed across the chamber with the central compartment receiving a continuous perfusion of oxygenated Krebs' solution (2 mL/min). The temperature of the Krebs' solution was maintained at 37°C. The axons were stimulated at one end of the strip and the CAP was recorded at the opposite end.

### Treatments with pharmacological agents

L-glutamate sodium, kainic acid, 4-aminopyridine (4-AP), MK-801, and GYKI52466 hydrochloride (Sigma, St. Louis, MO) were directly dissolved in the Krebs' solution. N-methyl-D-aspartate (NMDA), NS-102, α-amino-3-hydroxy-5-methyl-4-isoxazolepropionic acid (AMPA), and calcium ionophore A23187, MDL 28170 (Sigma) were first dissolved in DMSO and then diluted with Krebs' solution to a final desired concentration. The incubations were carried out at room temperature except the examinations on glutamate concentrations and glutamate receptor antagonists.

### Immunohistochemistry

 Antiserum against degenerated myelin basic protein (MBP) (Millipore Corp. Billerica, MA) was used to examine the myelin damage in the spinal cord white matter. This antibody stains myelin only in damaged, but not intact, white matter regions [13], [20]. Antiserum against Kv1.2 antibody (Alomone Lab, Jerusalem, Israel) was used to locate the K^+^ channels at the juxtaparanodes. The immunofluorescence images were taken by TPEF or confocal fluorescence on the same laser-scanning microscope. A 543 nm He-Ne laser was used for confocal fluorescence imaging.

### Electron Microscopy

The ultrastructural change of myelin was characterized by transmission electron microscopy (TEM). Both the glutamate-treated and the control tissues were immersed into a fixative solution (3% glutaraldehyde in 0.1 M cold cacodylate buffer containing 2 mM MgCl_2_, 1 mM CaCl_2_, 0.25% NaCl, pH = 7.4) for 10 min. The tissues were then dissected into small pieces and fixed for an additional 80 min. The dissected samples were rinsed with 0.1 M cacodylate buffer three times and deionized water one time, and then post-fixed in aqueous reduced osmium (1% OsO_4_ and 1.5% K_3_Fe(CN)_6_) for 90 min at room temperature. After washing three times with deionized water, the fixed specimens were dehydrated through a graded ethanol series, embedded in Epon, and polymerized at 60°C for 48 h. Finally, the specimens were dissected into thin sections (90–100 nm), stained with uranyl acetate and lead citrate, and imaged on an FEI/Philips CM-10 bio-twin transmission electron microscope.

### Statistical analysis

 The statistical data were presented as mean±s.e.m. A paired Student's *t*-test was used to compare measurements between two groups. ANOVA with Tukey's test was used for multiple comparisons within a group. For CAP measurements, *n* represents the number of individual spinal white matter strips. For quantification of ratios of nodal length to nodal diameter, *n* represents the number of nodes analyzed for ratio calculations. The data in one group were obtained from at least six spinal cord white matter strips which came from three animals. For imaging of one spinal cord strip, we first randomly chose one position as the starting point. Then, we acquired CARS images of every node we found by moving the stage with raster-scanning. We usually took 15–30 images of nodes per white matter strip and at least 100 images of nodes per group.

## Results

### CARS imaging reveals paranodal myelin splitting and retraction induced by glutamate

**Figure 1:**
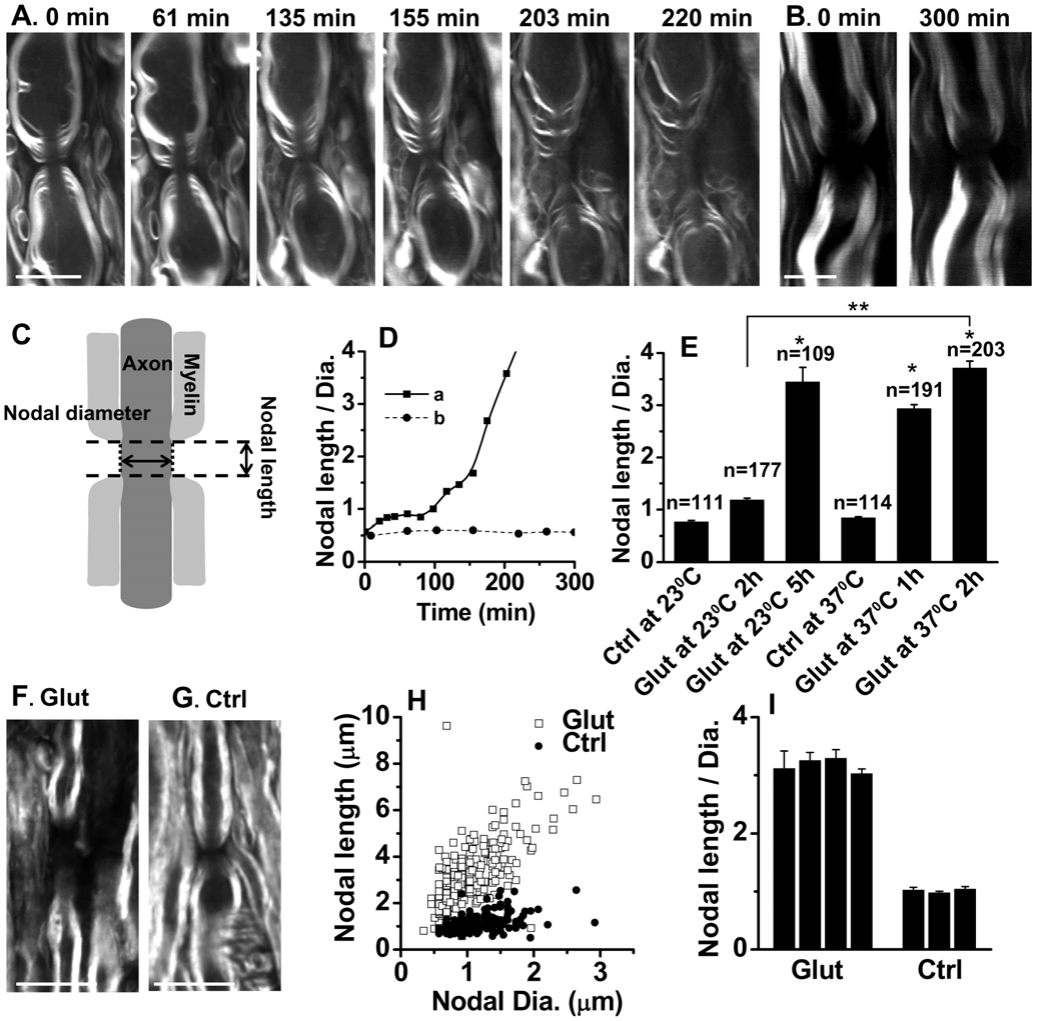
Real-time CARS imaging of paranodal myelin splitting and retraction induced by glutamate in spinal cord white matter. (A) CARS images of paranodal myelin after different periods of 1 mM glutamate treatment *ex vivo*. (B) CARS images of paranodal myelin in the control sample *ex vivo* without glutamate treatment. Little change was observed in paranodal myelin after 300 min. (C) Schematic nodal length and nodal diameter. (D) Changes of the nodal length-to-diameter ratio with time for the nodes in (A) and (B). (E) Temperature effect on the ratios of nodal length to diameter. * *p*<0.001 compared with the ‘Ctrl’ group at the corresponding temperature. ** *p*<0.001 between two indicated groups. (F) Typical CARS image of paranodal myelin in the rat spinal cord after 12 h of *in vivo* 1 mM glutamate treatment. (G) Typical CARS image of paranodal myelin in the rat spinal cord after 12 h of *in vivo* saline treatment. (H) With similar nodal diameters, the *in vivo* glutamate-treated group (Glut) shows larger nodal length than the *in vivo* saline-treated group (Ctrl). (I) The *in vivo* glutamate-treated group has 3 times larger ratios of nodal length to nodal diameter than the *in vivo* saline-treated group. Each column represents the mean ratio of nodal length to nodal diameter measured from one rat. Bar = 10 µm.

 The response of paranodal myelin to glutamate application was visualized on a laser-scanning CARS microscope. A typical myelin structure around a node of Ranvier in an *ex vivo* spinal ventral column is shown in Figure 1A. At 0 min, the paranodal myelin loops adjacent to a node were clearly resolved by the CARS contrast from symmetric CH_2_ stretch vibration [17]. Myelin degradation around the node following 1 mM glutamate application was monitored in real time. At about 60 min, paranodal myelin was observed to split originating from paranodal loops and extending toward internodes. Along with splitting the paranodal myelin retracted away from the node, leading to significant exposure of the axon after 200 min. In the control sample without glutamate treatment, neither myelin splitting nor retraction was observed during 5 h incubation with oxygenated Krebs' solution (Figure 1B).

To quantify the retraction of paranodal myelin from axons of different diameters, we measured the ratio of nodal length to nodal diameter at different time points of post-glutamate treatment. As shown in Figure 1C, the nodal length is defined as the distance between paranodal myelin at two ends along the node, which includes the length of both node and additionally exposed axon during paranodal myelin retraction. The nodal diameter is defined as the distance between paranodal myelin at two sides across the node. In the control sample without glutamate treatment, the nodal length-to-diameter ratio was measured as 0.76±0.03 (*n* = 111). Incubation with 1 mM glutamate for 300 min increased the ratio to 3.45±0.29 (*n* = 109). For the specific node shown in Figure 1A, the application of glutamate increased the ratio by more than 4 times in 220 min (Figure 1D).

 The extent of white matter injury is highly sensitive to variation of temperature [21], [22]. Our real-time CARS imaging was performed at room temperature of 23°C due to the use of water objective. To compare the structural changes of paranodal myelin induced by glutamate at physiological temperature of 37°C and at room temperature of 23°C, we incubated the spinal tissues with 1.0 mM glutamate for 2 h at 23°C, 5 h at 23°C, 1 h at 37°C, and 2 h at 37°C, respectively. We observed the same structural changes, splitting and retraction of paranodal myelin, at 37°C (Supplementary Figure S3), but with an increased speed. At the same incubation time of 2 h, the ratio of nodal length to diameter at 37°C is significantly higher than that at 23°C (Figure 1E). The ratio obtained with 2-hour incubation at 37°C is close to that obtained with 5-hour incubation at 23°C. These results indicate that glutamate induces the same kind of paranodal myelin damage at an accelerated rate under physiological temperature, possibly due to more efficient enzymatic activities.

To explore the effect of glutamate on paranodal myelin under a more physiological condition, *in vivo* glutamate treatment was performed on the spinal cord of Long-Evans rats. In the extracted spinal cord after in vivo treatment (Glut), paranodal myelin retraction was extensively observed (Figure 1F). In the control group with saline treatment (Ctrl), paranodes in the spinal cord were tightly covered by myelin (Figure 1G). The measurements of nodal lengths and nodal diameters (Supplementary Table S1) showed that with similar nodal diameters the ‘Glut’ group at the exposed site displayed larger nodal lengths (Figure 1H) than the ‘Ctrl’ group. And the ratio of nodal length to diameters was about 3 times larger than that of the ‘Ctrl’ group (Figure 1I). These results demonstrate that glutamate could inflict paranodal myelin retraction in the spinal cord of live animals.

### Glutamate breaks axo-glial junctions and disrupts the paranodal myelin

**Figure 2:**
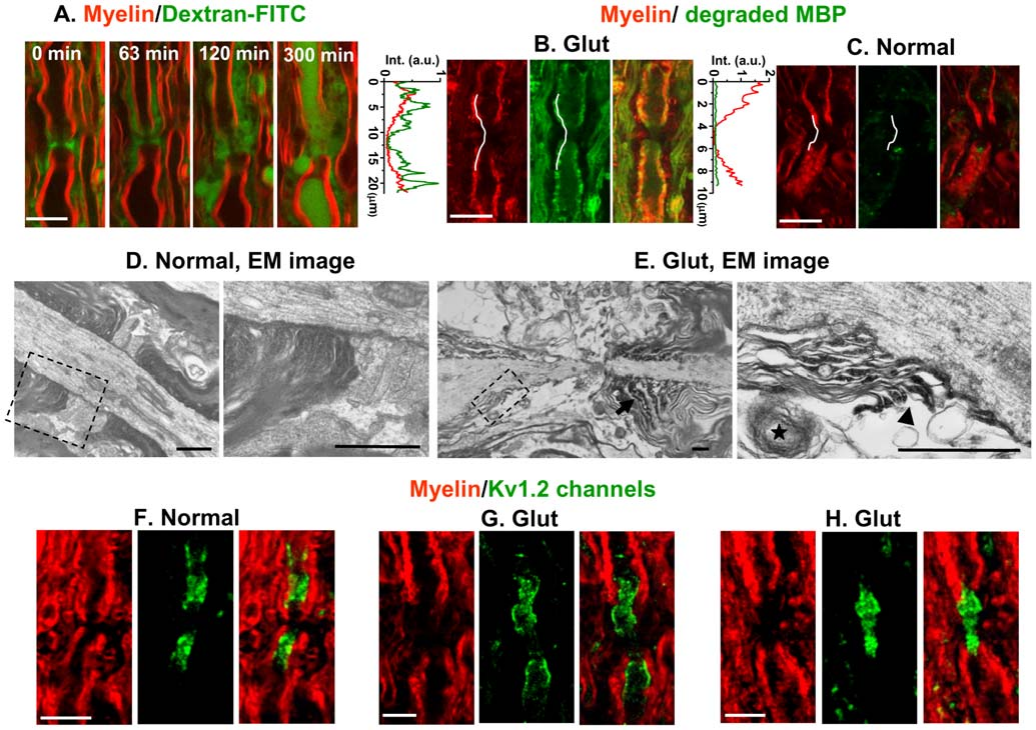
Glutamate application breaks axo-glial junctions, disrupts paranodal myelin and subsequently exposes juxtaparanodal K^+^ channels. (A) Time-lapse images showing the leakage of dextran-FITC into the split myelin after glutamate application. The myelin (red) and dextran-FITC (green) were monitored by CARS and TPEF, simultaneously. (B) and (C) CARS images of myelin sheath (red) and confocal fluorescence images of degraded MBP (green) in spinal tissues after incubation in 1 mM glutamate solution (Glut) or normal Krebs' solution (Normal). The curves on the left of the images are intensity profiles of the lines indicated in the images. (D) EM images in a normal tissue show the paranodal myelin held in tight contact with the axolemma. The right panel is the magnified image of the dash frame in the left panel. (E) EM images show that glutamate induces paranodal myelin splitting (arrow), disruption and retraction. The right panel is the magnified image of the dash frame in the left panel. An elongated node, detachment of paranodal myelin from axolemma (arrow head), and disrupted myelin debris (star) were observed. (F)–(H) CARS images of myelin sheath (red) and TPEF images of Kv1.2 channels (green) at the juxtaparanodes after normal Krebs' solution (F, Normal) and application of glutamate (G–H, Glut). Both the exposure of Kv1.2 channels (G) and displacement of Kv1.2 channels into paranodes and node (H) were observed. For (D) and (E), bar = 1 µm. For (A)–(C) and (F)–(H), bar = 10 µm.

 Normally the paranodal myelin is held on the axolemma by the axo-glial junctions which segregate the Na^+^ and K^+^ channels along the axolemma [23]. The observed splitting and retraction of paranodal myelin by exposure to glutamate suggest the breakdown of the axo-glial junctions. To examine whether this process did occur, the spinal tissues were pre-incubated with Krebs' solution containing dextran-FITC (MW = 4,400) as a fluid phase marker for 1 h prior to glutamate exposure. The myelin (red) change and the dextran-FITC (green) distribution were simultaneously monitored by CARS and TPEF, as shown in Figure 2A. The dextran-FITC was initially kept in the extracellular space (0 min). With glutamate application that resulted in the paranodal myelin splitting, the dextran-FITC leaked into the split myelin (63 min) and eventually into the injured axon (300 min). In the control experiment where the spinal tissues were incubated with Krebs' solution for 5 h, no dextran-FITC accumulation was observed in the periaxonal space, myelin or axon. Hence, the diffusion of dextran-FITC into the split myelin clearly demonstrates that glutamate treatment resulted in the breakdown of axo-glial paranodal junctions. To determine whether this process is associated with myelin degradation, we further performed immunostaining of degraded MBP. In a glutamate-treated sample (Glut, ratio of nodal length to diameter = 3.5), the paranodal region shows a weak CARS signal (red) by the action of myelin splitting and retraction, whereas a relatively strong fluorescence signal (green) from degraded MBP was observed (Figure 2B). On the contrary, a control sample without glutamate application (Normal, ratio of nodal length to diameter = 1.3) displayed a much weaker signal of degraded MBP (Figure 2C). This result demonstrates that glutamate treatment resulted in degradation of paranodal myelin.

 The glutamate-induced breakdown of axo-glial junctions and paranodal myelin disruption were also supported by EM images (Figure 2D and 2E). In a glutamate-treated sample, we observed the rupture of junctions, paranodal myelin disruption and retraction (Figure 2E), which were characterized by the detachment of paranodal myelin from axolemma, myelin debris and an elongated node, respectively.

###  Exposure and redistribution of juxtaparanodal K^+^ channels

Paranodal myelin retraction is expected to expose K^+^ channels. It is known that a high density of voltage-gated K^+^ channels, especially Kv1.1, Kv1.2, and Kvβ2 subunits, are normally located at the juxtaparanodes and protected by myelin [24], [25]. The K^+^ channel distribution was examined by imaging of immunostained Kv1.2 channels (Figure 2F–2H). In normal samples (Figure 2F), the Kv1.2 channel (green) was concealed beneath the compact paranodal myelin (red). In glutamate-treated samples, disruption of paranodal myelin (CARS, red) and subsequent exposure of Kv1.2 channels (TPEF, green) (Figure 2G) were observed. Furthermore, 30.3% exposed potassium channels displayed the displacement of Kv1.2 channel to the paranodes and node (Figure 2H). The redistribution of K^+^ channel further demonstrates the breakdown of the axo-glial paranodal junctions.

### Glutamate exposure impairs spinal cord axonal conduction

**Figure 3:**
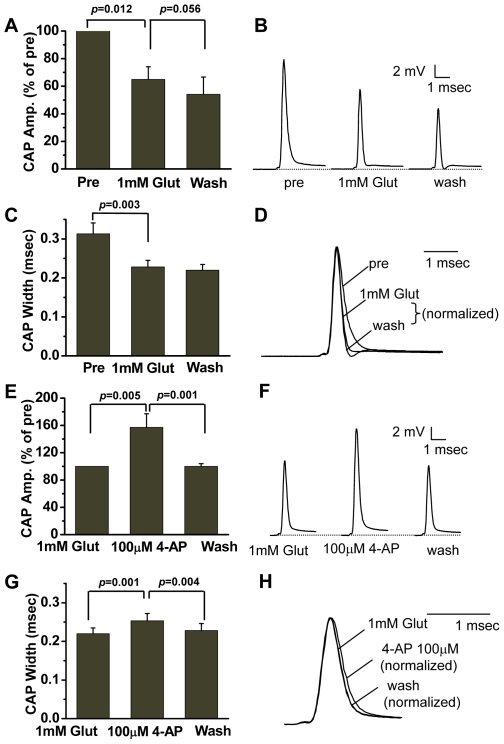
Effect of glutamate (Glut) and 4-AP on CAPs in *ex vivo* spinal ventral columns. (A) Histogram showing an irreversible increase in mean peak CAP amplitudes (normalized to 100% of the CAP amplitude (Pre) prior to glutamate treatment) after 1 mM glutamate treatment. (B) Representative CAP recordings before and after glutamate treatment and washing. (C) Histogram showing a decrease in CAP widths at half amplitude after glutamate treatment. (D) Representative CAP recordings showing a width decrease after glutamate treatment. The CAP amplitudes were normalized to the same level of pre-glutamate treatment (Pre) in order to compare CAP widths at half amplitude. (E) Histogram showing an increase in mean peak CAP amplitudes (normalized to 100% of the CAP amplitude (1 mM Glut) after glutamate treatment and washing) after 100 µM 4-AP treatment. (F) Representative CAP recordings before and after 4-AP treatment. (G) Histogram showing an increase in CAP widths at half amplitude after 4-AP treatment. (H) Representative CAP recordings showing a width increase after 4-AP treatment. The CAP amplitudes were normalized to the same level of that before 4-AP treatment (1 mM Glut, after glutamate treatment and washing) in order to compare CAP widths at half amplitude. In all cases, a paired Student's *t*-test was used to compare measurements between two groups (*n* = 6).

The juxtaparanodal K^+^ channels normally contribute little to axonal conduction, whereas their exposure following demyelination was shown to result in loss of axonal conduction [26]. Meanwhile, application of glutamate through the perfusion medium has been shown to cause a striking decrease of CAP amplitude in spinal dorsal columns [13]. To quantify glutamate toxicity on impulse conduction, a double sucrose gap chamber (Supplementary Figure S2) [19] was used to record CAP from a 4-cm ventral column from guinea pig spinal cord. It was found that the CAP amplitude declined to 65.0±9.2% of the initial value (Pre in Figure 3A and 3B) after exposure to 1 mM glutamate for 2 h. Washing with normal Krebs' solution for 1 h did not stop the decline in CAP amplitude, decreasing, instead, to 54.1±12.5%. Importantly, the CAP width, defined as the CAP trace width at half amplitude, reduced from 0.31±0.03 ms to 0.23±0.02 ms (Figure 3C and 3D) after 2 h of glutamate application. Washing with normal Krebs' solution for 1 h made no significant change in CAP width. In control spinal ventral columns perfused with normal oxygenated Krebs' solution, the CAPs showed little change during 4 h [27].

### Glutamate-induced axonal conduction deficit can be recovered by 4-AP

Application of 4-AP, a K^+^ channel blocking agent, has been shown to increase both CAP amplitude and width in focally demyelinated axons [28]. 4-AP has also been found to restore conduction of demyelinated pathways in acute and chronic spinal cord injury models [29], [30] and MS patients [31]. To examine its effect on glutamate-treated samples, we added 100 µM 4-AP to the perfusion solution following glutamate treatment. It was found that incubation of glutamate-treated spinal ventral columns with 4-AP for 1 h restored the CAP amplitude to 157±20% of the value after glutamate treatment/washing and before 4-AP application (Figure 3E and 3F). The 4-AP application also increased the CAP width from 0.23±0.02 ms to 0.25±0.02 ms (Figure 3G and 3H). After washing with normal Krebs' solution for 1 h, the CAP amplitude changed back to 100±4.1% of that before 4-AP application (Figure 3E) and the CAP width returned to 0.23±0.02 ms (Figure 3G). Application of 100 µM 4-AP to ventral columns without glutamate treatment did not significantly affect the CAP amplitude or width [30]. The reduction of CAP amplitude and width together with the 4-AP data supports that glutamate-induced functional loss is caused by the observed retraction of paranodal myelin and consequent exposure of K^+^ channels beneath the myelin.

### Calcium influx and calpain activation are involved in glutamate-induced paranodal myelin damage

**Figure 4:**
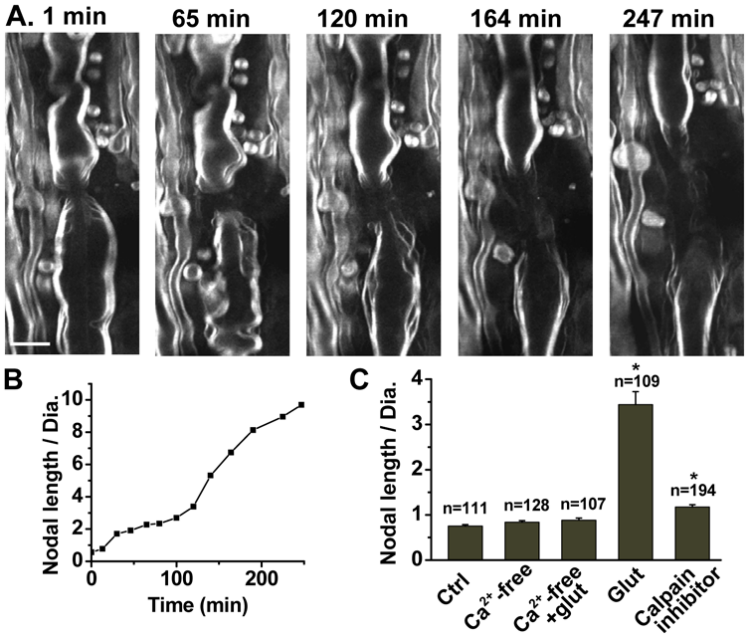
Ca^2+^ influx and calpain activation are involved in glutamate-induced paranodal myelin damage. (A) Time-lapse CARS images of paranodal myelin after different periods of treatment with 250 µg/mL Ca^2+^ ionophore A23187. Bar = 10 µm. (B) Increase of the nodal length-to-diameter ratio with time for the node in (A). (C) Statistical analysis of ratios of nodal length to nodal diameter under five different conditions. ‘Ctrl’ represents samples incubated in Krebs' solution. ‘Ca^2+^-free’ represents samples incubated in Ca^2+^-free Krebs' solution. ‘Ca^2+^-free + Glut’ represents samples exposed to 1 mM glutamate in Ca^2+^-free Krebs' solution. ‘Glut’ represents samples exposed to 1 mM glutamate in normal Krebs' solution. ‘Calpain inhibitor’ represents samples exposed to calpain inhibitor III MDL 28170 and glutamate together. Five groups of samples were exposed to the different conditions for 5 h. * *p*<0.001 between the ‘Glut’ group and the ‘Ctrl’ group, and between the ‘Calpain inhibitor’ group and the ‘Glut’ group.

Glutamate receptors have been shown to display Ca^2+^ permeability [32], and Ca^2+^ influx through these receptors contributes to neuronal death in several pathophysiological conditions, such as anoxia/ischemia and trauma [1]. To evaluate the role of Ca^2+^ influx in paranodal myelin damage, we incubated the spinal ventral column in oxygenated Krebs' solution supplemented with 250 µg/mL Ca^2+^ ionophore A23187. Similar to the results obtained from glutamate application, CARS imaging revealed paranodal myelin splitting and then retraction (Figure 4A), with the ratio of nodal length to diameter increasing by nearly 10 times in 250 min (Figure 4B).

The role of Ca^2+^ in glutamate-induced paranodal myelin damage was quantitatively studied by measuring the nodal length-to-diameter ratio in four groups of tissues. The samples in each group were incubated with normal Krebs' solution (Ctrl), Ca^2+^-free Krebs' solution (Ca^2+^-free), Ca^2+^-free Krebs' solution plus 1 mM glutamate (Ca^2+^-free + Glut), and normal Krebs' solution plus 1 mM glutamate (Glut), respectively. After 300 min incubation with different solutions, we measured the nodal length-to-diameter ratios in the CARS images. It was found that the ‘Glut’ group displayed an average nodal length-to-diameter ratio 4.54 times larger than the ‘Ctrl’ group, whereas both the ‘Ca^2+^-free’ and ‘Ca^2+^-free + Glut’ groups displayed no significant difference in ratios of nodal length to diameter compared with the ‘Ctrl’ group (Figure 4C). In other words, by exclusion of Ca^2+^ from the incubating solution, paranodal myelin damage was avoided, strongly suggesting that glutamate-induced paranodal myelin change is a Ca^2+^-dependent process.

We further examined the involvement of calpain in this process. Calpains represent a family of Ca^2+^-dependent proteinases which break down myelin through degrading myelin basic proteins [33]. The ventral spinal column was pre-incubated for 60 min with calpain inhibitor III MDL 28170, a broad cell-permeable calpain inhibitor. Then glutamate was added into the incubating solution to the final concentration of 1 mM. We found that MDL 28170 significantly reduced the glutamate-induced damage to paranodal myelin and the ratio of nodal length to diameter for MDL 28170-treated samples (‘Calpain inhibitor’ group in Figure 4C) decreased almost 3 times compared with the ‘Glut’ group. This result clearly shows that calpain plays an important role in the pathway of glutamate excitotoxicity.

### Glutamate excitotoxicity in paranodal myelin is mediated by kainate and NMDA receptors

**Figure 5:**
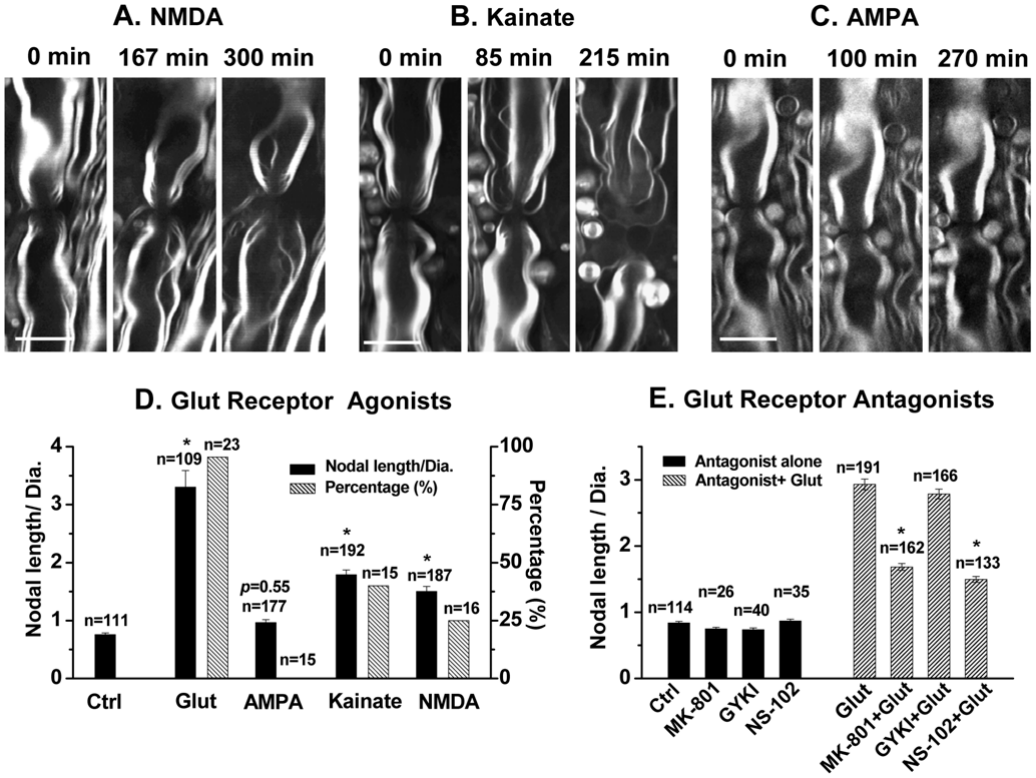
Glutamate excitotoxicity in paranodal myelin is mediated by kainate and NMDA receptors. (A) CARS images of paranodal myelin under NMDA (0.68 mM plus 27 µM glycine) treatment at different time periods. 27 µM glycine was used to maximize activation of NMDA receptors. (B) CARS images of paranodal myelin under kainate (1 mM) treatment at different time periods. (C) CARS images of paranodal myelin under AMPA (0.16 mM) treatment at different time periods. (D) NMDA, kainate, but not AMPA induced paranodal myelin retraction. Black bars represent the average ratios of nodal length to diameter under various treatments. White bars with patterns represent the percentages of paranodal myelin damage under various treatments. * *p*<0.001 compared with the control. (E) Glutamate-induced paranodal myelin retraction is partially diminished by antagonists of NMDA, kainate, but not AMPA receptor. MK-801 (a noncompetitive NMDA antagonist), GYKI52466 (a selective AMPA antagonist) or NS-102 (a selective kainate antagonist) were used. The spinal tissues were pre-incubated in the Krebs' solution supplemented with a specific glutamate receptor antagonist at 37°C for 0.5 h. Glutamate (1 mM) was then added into the incubating solution. After additional 1 h incubation, the nodal lengths and nodal diameters in the spinal tissue were measured by CARS. * *p*<0.001 compared with the ‘Glut’ group. Bar = 10 µm in (A–C).

It has been shown that NMDA receptors are expressed in the developing and mature CNS myelin and play a crucial role in ischemic injury [34], [35], [36]. On the other hand, previous studies have also shown that blockade of AMPA/kainate, but not NMDA receptors, provided functional and histological protection against ischemia [37], [38]. To determine what subtypes of glutamate receptors are involved in glutamate-induced paranodal myelin damage, we carried out CARS imaging of spinal tissues after 5-hour incubation with Krebs' solutions containing the glutamate receptor agonists NMDA, AMPA, and kainate, respectively. The splitting and retraction of paranodal myelin, characterized by an increase of the ratio of nodal length to diameter by more than 2 times, were observed in 25% of spinal tissues (*n* = 16) treated with 0.68 mM NMDA and 40% of spinal tissues (*n* = 15) treated with 1 mM kainate, but none in spinal tissues (0%, *n* = 15) treated with 0.16 mM AMPA. Typical imaging results are shown in Figure 5A, 5B and 5C, respectively.

We also measured the ratios of nodal length to diameter after 5-hour treatment of spinal tissues at room temperature with NMDA, AMPA and kainate, respectively. As shown in Figure 5D, the nodal length-to-diameter ratios for the NMDA- and kainate-treated groups were 1.49±0.09 (*n* = 187) and 1.79±0.08 (*n* = 192), respectively, which showed significant difference (*p<0.001) in comparison with the control group (0.76±0.03) without any agonist. On the other hand, the ratio of 0.96±0.05 (*n* = 177) for the AMPA-treated group did not significantly differ from the control group (*p*>0.05), consistent with the observation that no paranodal myelin retraction happened with AMPA treatment (Figure 5C). The role of NMDA and kainate receptors was confirmed by applications of a noncompetitive NMDA receptor antagonist MK-801, a selective AMPA receptor antagonist GYKI52466, and a selective kainate receptor antagonist NS-102, respectively. As shown in Figure 5E, co-application of MK-801 (10 µM) or NS-102 (20 µM) with glutamate (1 mM) partially protected the paranodal myelin from the glutamate excitotoxicity. Correspondingly, the ratios of nodal length to diameter were reduced by nearly 45%. In contrast, co-application of GYKI52466 (30 µM) with glutamate did not prevent the paranodal myelin retraction. As a control, no paranodal myelin damage was observed with applications of individual antagonists. In summary, these results demonstrate that paranodal myelin damage induced by glutamate is mainly mediated by NMDA and kainate receptors.

### Paranodal myelin retraction precedes axonal injury

**Figure 6:**
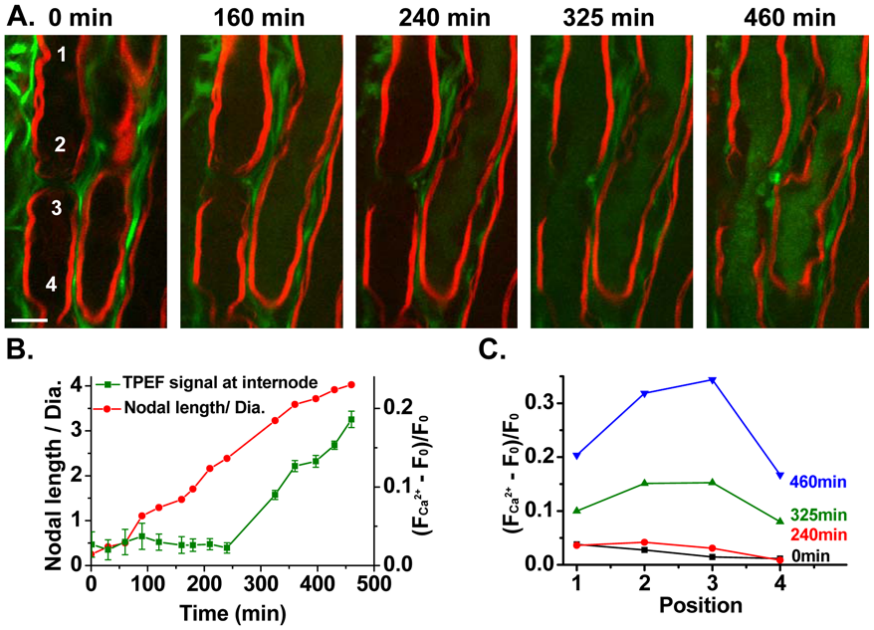
Paranodal myelin retraction precedes axonal injury. (A) Simultaneously acquired CARS image of myelin sheath (red) and TPEF image of a calcium indicator, Oregon green 488 AM (green), including a node of Ranvier during glutamate treatment. The Ca^2+^ influx observed at 240 min was preceded by paranodal myelin splitting and retraction, which began at 60 min of glutamate treatment. Bar = 10 µm. (B) Ratios of nodal length to diameter for the node in (A) and intra-axonal TPEF signals recorded at different time points. F_Ca_^2+^ represents the TPEF intensity from Ca^2+^ indicator Oregon green 488 AM. F_0_ is the background intensity. (C) The TPEF signal of Oregon green 488 AM at different positions marked in (A, 0 min) varied after glutamate application for 0 min (black), 240 min (red), 325 min (green) and 460 min (blue), respectively. The signal elevation trends at the four positions implicate that Ca^2+^ conceivably flew into axons via the exposed paranodes and juxtaparanodes and then spread into the internodal area.

 As a consequence of the severe retraction of paranodal myelin, the axon cylinder is directly exposed to the ambient glutamate. To examine axonal injury, we pre-incubated the spinal tissues with a cell-permeable Ca^2+^ indicator, Oregon Green 488 AM BAPTA-2, in a Ca^2+^-free Krebs' solution for 2 h. After washing with normal Krebs' solution, we treated the spinal tissue with 1 mM glutamate and monitored the paranodal myelin by CARS and Ca^2+^ by TPEF simultaneously. Before glutamate application (0 min), the TPEF signal mainly arose from the fibrous structures between myelinated axons (Figure 6A). These fibrous structures were shown to be astrocyte processes in the white matter [39]. The intracellular Ca^2+^ level in the axon was observed to start increasing at 240 min (Figure 6B). In contrast, paranodal myelin had significantly retracted at 160 min, and by 240 min had retracted to a nodal length-to-diameter ratio of 2.4 (Figure 6B). In the control sample without glutamate treatment, the axon cylinders did not show any detectable signal of Oregon Green 488 AM (data not shown). These results demonstrate that Ca^2+^ influx into the axon occurred after paranodal myelin damage. The intra-axonal TPEF intensity at different positions (indicated by numbers in Figure 6A) was further measured to explore the route of Ca^2+^ influx into the axon. The intensity levels after different time periods of glutamate application are shown in Figure 6C. The elevation of Ca^2+^ concentration was faster at the exposed paranodes and juxtaparanodes (positions 2 and 3) than that at the internodes (positions 1 and 4). Therefore, it is conceivable that Ca^2+^ first entered the axons through the exposed paranodes and juxtaparanodes and then spread to the internodes along the axon cylinders.

## Discussion

Growing evidence has shown that glutamate, a principal CNS excitatory neurotransmitter, plays a key role in the pathology of white matter diseases [9], [40]. Elevated glutamate level was detected in the normal-appearing white matter and acute contrast-enhancing lesions of MS patients by magnetic resonance spectroscopy [41]. Increased glutaminase (glutamate-producing enzyme) expression in activated immune cells was found in active MS lesions [3]. Furthermore, glutamate receptor antagonists were found to reduce neurological deficits in experimental autoimmune encephalomyelitis, an animal model of MS [42], [43]. Elevated extracellular glutamate level was shown to result in the death of oligodendrocytes [11], [12] and neurons [44], [45] through excitotoxic mechanisms [40]. However, the role of glutamate in demyelination is not fully understood. In this study, the application of CARS microscopy, a vibrational imaging tool with 3D sub-micron resolution, allowed direct visualization of paranodal myelin damage following glutamate application in spinal cord *ex vivo* and *in vivo*. Paranodal myelin was found to be extremely vulnerable to glutamate exposure at the concentration of 1 mM or 0.1 mM (Supplementary Figure S4). Paranodal myelin loops were observed to split and retract towards the internode (Figure 1A). The damage was associated with breakdown of axo-glial junctions (Figure 2A and 2E), disruption of paranodal myelin (Figure 2B and 2E), and exposure and redistribution of juxtaparanodal K^+^ channels (Figure 2G and 2H).

The observed exposure and redistribution of K^+^ channels was supported by CAP recording of conduction impairment induced by glutamate. The exposure of K^+^ channels located at the juxtaparanodes leads to excessive conductance of K^+^ channels so that the axolemma is held close to the equilibrium potential of K^+^. This shunting of local circuit currents through the K^+^ channels could impair the generation of action potentials, leading to the reduction of the CAP amplitude (Figure 3A). For the same reason, exposure of the K^+^ channels could cause the faster repolarization, which resulted in the decrease of the CAP width (Figure 3C). The exposure of K^+^ channels under the juxtaparanodes was further confirmed by the application of 4-AP, a K^+^ channel blocker which increased both the CAP amplitude and width (Figure 3E and 3G) after glutamate-induced conduction deficit. Although the internodal myelin damage might also contribute to the impaired conduction [13], our results suggest that the disruption of paranodal myelin induced by glutamate is an important mechanism for the functional loss in the white matter injury. Notably, 4-AP is currently undergoing clinical trials in patients with MS or chronic spinal cord injury [28]. Our observations provide direct visual evidence to support 4-AP as a potential drug for the treatment of demyelinating disorders.

The role of AMPA, kainate and NMDA receptors in white matter injury has been extensively examined. Based on studies on cultured oligodendrocytes, AMPA and kainate receptors are located on oligodendrocytes and they mediate cell death [11], [12]. Further studies using isolated white matter showed that blockade of AMPA receptors, not NMDA receptors provided functional or histological protection against ischemia in adult mouse brain slice [14], ischemia in mouse optic nerve [37], [46], and glutamate application in dorsal spinal cord of adult rats [13]. The treatment of AMPA/kainate antagonist NBQX was demonstrated to prevent oligodendrocyte death and axonal damage in experimental allergic encephalomyelitis [42]. *In vivo* treatment of NMDA receptor antagonist MK-801 failed to protect myelinated axons after focal cerebral ischemia [47]. Recent studies however revealed that functional NMDA receptors are expressed on myelin sheath in the white matter of the developing and adult CNS tissues including rat cerebellum and corpus callosum [34], and the mouse and rat optic nerve [35], [36], [37], suggesting that the presence of NMDA receptors is a common feature of myelin sheath regardless of its maturation stage [48]. Interestingly, NMDA receptors on myelin were observed to mediate Ca^2+^ accumulation [36] and cause myelin loss [35] during chemical ischemia. Because AMPA receptors are mainly located on oligodendrocyte somata, blockade of NMDA receptors, not AMPA receptors prevented the injury to myelin sheath [35]. *In vivo* treatment of NMDA receptor antagonist CNS 1102 following temporary focal ischemia was demonstrated to prevent myelin sheath loss in the cerebral white matter from ischemic injury [49]. In the current work, we found that NMDA and kainate, but not AMPA were able to partially induce paranodal myelin damage in isolated ventral spinal cord white matter from adult guinea pig. This observation suggests that in our model the paranodal myelin damage induced by glutamate may be mediated by NMDA and kainate receptors located on the paranodal myelin [50].

Our study further revealed the important role of calpain in glutamate-induced paranodal myelin damage. Calpain activation has been demonstrated to cleave myelin proteins including myelin basic protein and myelin-associated glycoprotein [51] and contribute to myelin disruption in animal models for spinal cord injury [52] and MS [53]. Glutamate excitotoxicity is known to play a major role in white matter injury of these disease models. However, whether myelin damage induced by the elevated glutamate level attributes to the activation of calpain has not been examined. Our result shows that calpain inhibitor can effectively reduce the paranodal myelin retraction when spinal tissue was exposed to high concentration of glutamate and thus demonstrates that calpain activation is essential for the process of glutamate-induced myelin damage.

Calpain activation degrades myelin proteins such as MBP and subsequently disrupts the structural integrity of myelin sheath. In the paranodal axo-glial junctions, the oligodendrocyte transmembrane protein Neurofascin Nf155 connects with a complex of paranodin/contactin-associated protein (Caspr) and contactin at the axolemma [54], [55]. It is therefore possible that the calpain activation might disrupt the transmembrane protein Nf155 and subsequently break the adhesion junctions between axolemma and paranodal myelin. In accordance with our result, elongated paranodal expression and disruption of Neurofascin Nf155 were observed in MS demyelinating lesions [7]. Similar structural changes of paranodal myelin implicate the possible involvement of glutamate-induced paranodal myelin disruption in the demyelination process of MS patients. Notably, because Ca^2+^ influx into axons was observed to follow the paranodal myelin retraction (Figure 6A), our data have excluded the possibility of breaking adhesion junctions from the axonal side. The intra-axonal integrity after 180 min of glutamate exposure was previously observed by immunostaining of spectrin breakdown products [13].

The current work shows great potentials of CARS microscopy in the study of white matter injury. First, CARS microscopy allows label-free and real-time imaging of myelin sheaths in both healthy and diseased states. Its 3D sub-micron resolution enables monitoring of the detailed myelin changes around nodes of Ranvier. Second, other nonlinear optical imaging techniques can be integrated into a CARS microscopic platform for studying multiple components in the white matter. For example, TPEF can be incorporated to study calcium activity with the aid of a calcium indicator. Sum frequency generation can be used to visualize astroglial filaments in astrocyte processes [39]. Third, the label-free molecular imaging capability makes CARS microscopy appealing for *in vivo* study of myelin by avoiding the inefficient diffusion and nonspecific binding of fluorophore probes in the tissue environment [56]. Together, these capabilities open up new opportunities in understanding the molecular mechanisms and cellular pathways in demyelinating diseases.

## Supporting Information

Figure S1 CARS energy diagram and imaging setup. (A) Energy diagram of CARS. In a CARS process, two laser fields at the pump (ω_p_) and Stokes (ω_s_) frequencies interact with a medium to generate a new field at the anti-Stokes frequency ω_as_  =  (ω_p_−ω_s_)+ω_p_. The CARS signal can be significantly enhanced when the beating frequency, (ω_p_−ω_s_), is in resonance with a molecular vibration. The coherent property leads the CARS signal to increase quadratically with respect to the number of vibrational oscillators in the focal volume. (B) CARS imaging setup. The two laser beams at frequencies ω_p_ and ω_s_ were generated from two tightly synchronized Ti:sapphire lasers (Mira 900/Sync-lock, Coherent Inc.). Both lasers have a pulse duration of 2.5 ps. The two beams were parallel polarized and collinearly combined. A Pockels' cell was used to reduce the repetition rate from 78 MHz to 7.8 MHz. The overlapped beams were directed into a laser scanning microscope (FV300/IX70, Olympus Inc.) and focused into a sample through a 60X water immersion objective lens (NA = 1.2). The CARS signal can be detected by forward- and epi- photomultiplier tube (PMT).
(0.20 MB PDF)

Figure S2 A double sucrose gap chamber for CAP recording. Briefly, a strip of spinal cord ventral white matter approximately 40 mm in length was placed across the chamber with the central compartment receiving a continuous perfusion of oxygenated Krebs' solution (2 mL/min). The stimulating and recording electrodes were not in direct contact with the spinal cord tissue. The temperature of the Krebs' solution was maintained at 37°C. The free ends of the white matter strip were placed across the sucrose gap channels to side compartments filled with isotonic (120 mM) potassium chloride. The sucrose gap was perfused with isotonic sucrose solution at a rate of 1 mL/min. The white matter strip was sealed with a thin plastic sheet and vacuum grease on either side of the sucrose gap channels to prevent the exchange of solutions. The axons were stimulated at one end of the strip and the CAP was recorded at the opposite end.
(0.06 MB PDF)

Figure S3 Temperature effect on glutamate-induced paranodal myelin retraction. (A) CARS image of a node of Ranvier (indicated by arrow) when the spinal tissue was incubated in Krebs' solution at 37°C for 2 h. (B) CARS image showing the retraction of paranodal myelin (indicated by arrow) when the spinal tissue was incubated with 1 mM glutamate at 37°C for 1 h. Bar = 10 µm.
(0.16 MB PDF)

Figure S4 Concentration effect on the ratios of nodal length to diameter. After spinal tissues were incubated with 0.1 mM glutamate at 37°C for 2 h, the retraction of paranodal myelin was observed and the ratio of nodal length to diameter was significantly increased in comparison with the ‘Ctrl’ group (without glutamate treatment). However, compared with the ratio of the 1.0 mM glutamate group, the use of a lower concentration significantly reduced the damage. Moreover, when the incubation time was extended from 2 h to 5 h, the ratio did not increase. These results indicate that glutamate-induced paranodal myelin damage is a concentration-dependent process. * Significant difference at p<0.001 level compared with the ‘Ctrl’ group.
(0.01 MB PDF)

Table S1 Analysis of ratios of nodal length to nodal diameter in spinal cord treated with glutamate *in vivo*.
(0.03 MB PDF)
