# Supplementary material for: Glutamate Excitotoxicity Inflicts Paranodal Myelin Splitting and Retraction
Source: PLoS One. 2009 Aug 20;4(8):e6705. doi: 10.1371/journal.pone.0006705 (PMC2725320; doi:10.1371/journal.pone.0006705)
Supplement: Figure S1 — CARS energy diagram and imaging setup. (A) Energy diagram of CARS. In a CARS process, two laser fields at the pump (ωp) and Stokes (ωs) frequencies interact with a medium to generate a new field at the anti-Stokes frequency ωas = (ωp−ωs)+ωp. The CARS signal can be significantly enhanced when the beating frequency, (ωp−ωs), is in resonance with a molecular vibration. The coherent property leads the CARS signal to increase quadratically with respect to the number of vibrational oscillators in the focal volume. (B) CARS imaging setup. The two laser beams at frequencies ωp and ωs were generated from two tightly synchronized Ti:sapphire lasers (Mira 900/Sync-lock, Coherent Inc.). Both lasers have a pulse duration of 2.5 ps. The two beams were parallel polarized and collinearly combined. A Pockels' cell was used to reduce the repetition rate from 78 MHz to 7.8 MHz. The overlapped beams were directed into a laser scanning microscope (FV300/IX70, Olympus Inc.) and focused into a sample through a 60X water immersion objective lens (NA = 1.2). The CARS signal can be detected by forward- and epi- photomultiplier tube (PMT). (0.20 MB PDF) [file pone.0006705.s001.pdf]

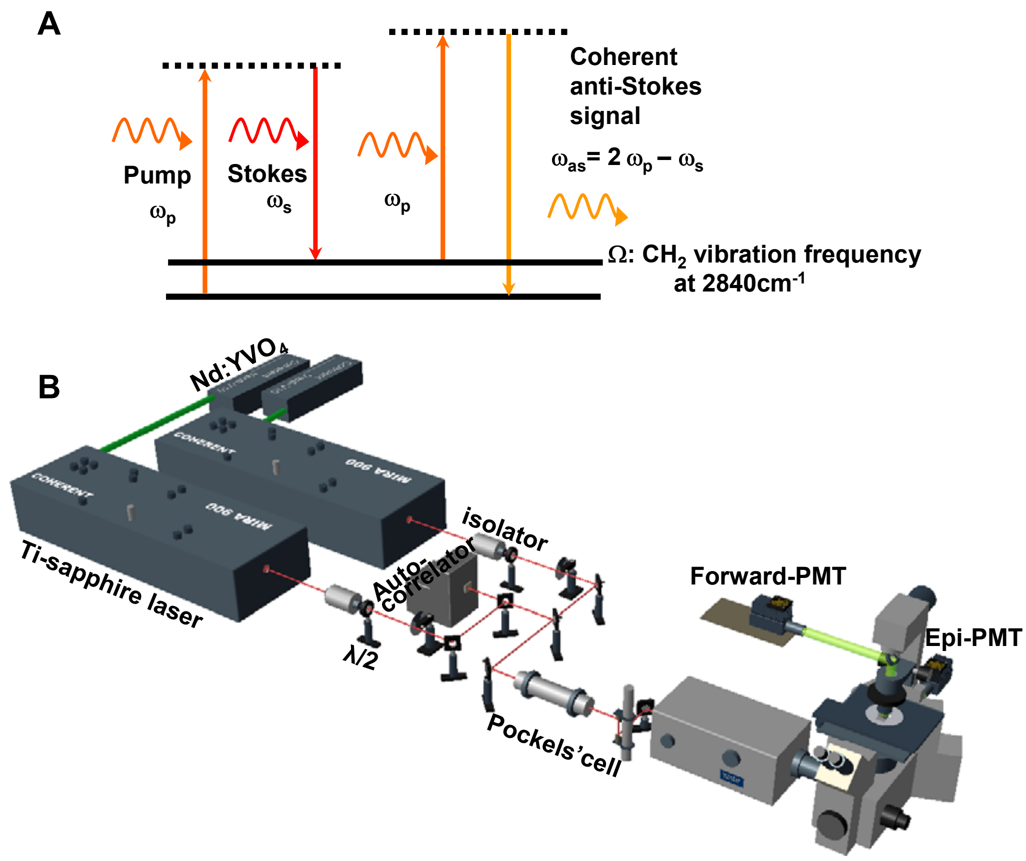

Figure S1. CARS energy diagram and imaging setup. (A) Energy diagram of CARS. In a CARS process, two laser fields at the pump (ω_p) and Stokes (ω_s) frequencies interact with a medium to generate a new field at the anti-Stokes frequency $\omega_{as} = (\omega_p - \omega_s) + \omega_p$. The CARS signal can be significantly enhanced when the beating frequency, $(\omega_p - \omega_s)$, is in resonance with a molecular vibration. The coherent property leads the CARS signal to increase quadratically with respect to the number of vibrational oscillators in the focal volume. (B) CARS imaging setup. The two laser beams at frequencies ω_p and ω_s were generated from two tightly synchronized Ti:sapphire lasers (Mira 900 / Sync-lock, Coherent Inc.). Both lasers have a pulse duration of 2.5 ps. The two beams were parallel polarized and collinearly combined. A Pockels' cell was used to reduce the repetition rate from 78 MHz to 7.8 MHz. The overlapped beams were directed into a laser scanning microscope (FV300/IX70, Olympus Inc.) and focused into a sample through a 60X water immersion objective lens (NA = 1.2). The CARS signal can be detected by forward- and epi- photomultiplier tube (PMT).
