# Supplementary material for: Glutamate Excitotoxicity Inflicts Paranodal Myelin Splitting and Retraction
Source: PLoS One. 2009 Aug 20;4(8):e6705. doi: 10.1371/journal.pone.0006705 (PMC2725320; doi:10.1371/journal.pone.0006705)
Supplement: Figure S2 — A double sucrose gap chamber for CAP recording. Briefly, a strip of spinal cord ventral white matter approximately 40 mm in length was placed across the chamber with the central compartment receiving a continuous perfusion of oxygenated Krebs' solution (2 mL/min). The stimulating and recording electrodes were not in direct contact with the spinal cord tissue. The temperature of the Krebs' solution was maintained at 37°C. The free ends of the white matter strip were placed across the sucrose gap channels to side compartments filled with isotonic (120 mM) potassium chloride. The sucrose gap was perfused with isotonic sucrose solution at a rate of 1 mL/min. The white matter strip was sealed with a thin plastic sheet and vacuum grease on either side of the sucrose gap channels to prevent the exchange of solutions. The axons were stimulated at one end of the strip and the CAP was recorded at the opposite end. (0.06 MB PDF) [file pone.0006705.s002.pdf]

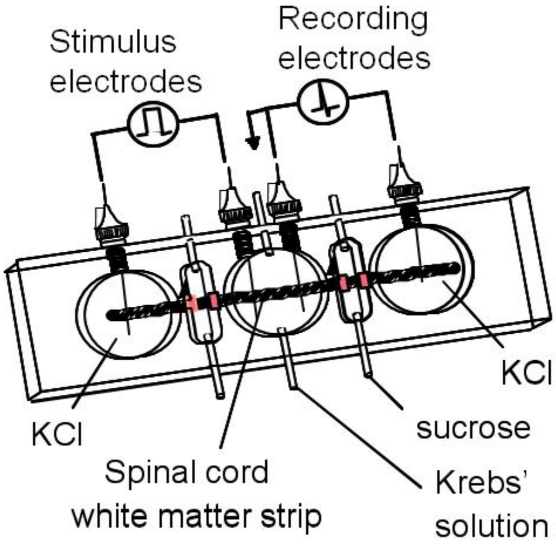

Figure S2. A double sucrose gap chamber for CAP recording. Briefly, a strip of spinal cord ventral white matter approximately 40 mm in length was placed across the chamber with the central compartment receiving a continuous perfusion of oxygenated Krebs' solution (2 mL/min). The stimulating and recording electrodes were not in direct contact with the spinal cord tissue. The temperature of the Krebs' solution was maintained at 37˚C. The free ends of the white matter strip were placed across the sucrose gap channels to side compartments filled with isotonic (120 mM) potassium chloride. The sucrose gap was perfused with isotonic sucrose solution at a rate of 1 mL/min. The white matter strip was sealed with a thin plastic sheet and vacuum grease on either side of the sucrose gap channels to prevent the exchange of solutions. The axons were stimulated at one end of the strip and the CAP was recorded at the opposite end.
