# Supplementary material for: Glutamate Excitotoxicity Inflicts Paranodal Myelin Splitting and Retraction
Source: PLoS One. 2009 Aug 20;4(8):e6705. doi: 10.1371/journal.pone.0006705 (PMC2725320; doi:10.1371/journal.pone.0006705)
Supplement: Figure S3 — Temperature effect on glutamate-induced paranodal myelin retraction. (A) CARS image of a node of Ranvier (indicated by arrow) when the spinal tissue was incubated in Krebs' solution at 37°C for 2 h. (B) CARS image showing the retraction of paranodal myelin (indicated by arrow) when the spinal tissue was incubated with 1 mM glutamate at 37°C for 1 h. Bar = 10 µm. (0.16 MB PDF) [file pone.0006705.s003.pdf]

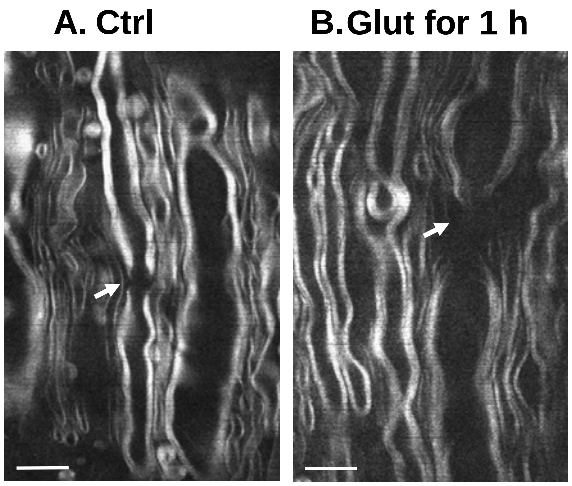

Figure S3. **Temperature effect on glutamate-induced paranodal myelin retraction.** (A) CARS image of a node of Ranvier (indicated by arrow) when the spinal tissue was incubated in Krebs' solution at 37 ºC for 2 h. (B) CARS image showing the retraction of paranodal myelin (indicated by arrow) when the spinal tissue was incubated with 1 mM glutamate at 37 ºC for 1 h. Bar = 10 μm.
