# Supplementary material for: Glutamate Excitotoxicity Inflicts Paranodal Myelin Splitting and Retraction
Source: PLoS One. 2009 Aug 20;4(8):e6705. doi: 10.1371/journal.pone.0006705 (PMC2725320; doi:10.1371/journal.pone.0006705)
Supplement: Figure S4 — Concentration effect on the ratios of nodal length to diameter. After spinal tissues were incubated with 0.1 mM glutamate at 37°C for 2 h, the retraction of paranodal myelin was observed and the ratio of nodal length to diameter was significantly increased in comparison with the ‘Ctrl’ group (without glutamate treatment). However, compared with the ratio of the 1.0 mM glutamate group, the use of a lower concentration significantly reduced the damage. Moreover, when the incubation time was extended from 2 h to 5 h, the ratio did not increase. These results indicate that glutamate-induced paranodal myelin damage is a concentration-dependent process. * Significant difference at p<0.001 level compared with the ‘Ctrl’ group. (0.01 MB PDF) [file pone.0006705.s004.pdf]

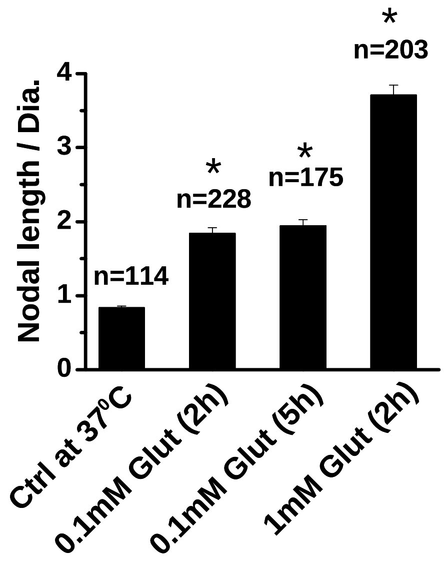

Figure S4. Concentration effect on the ratios of nodal length to diameter. After spinal tissues were incubated with 0.1 mM glutamate at 37 ºC for 2 h, the retraction of paranodal myelin was observed and the ratio of nodal length to diameter was significantly increased in comparison with the 'Ctrl' group (without glutamate treatment). However, compared with the ratio of the 1.0 mM glutamate group, the use of a lower concentration significantly reduced the damage. Moreover, when the incubation time was extended from 2 h to 5 h, the ratio did not increase. These results indicate that glutamate-induced paranodal myelin damage is a concentration-dependent process. * Significant difference at $p < 0.001$ level compared with the 'Ctrl' group.
