# Supplementary material for: Glutamate Excitotoxicity Inflicts Paranodal Myelin Splitting and Retraction
Source: PLoS One. 2009 Aug 20;4(8):e6705. doi: 10.1371/journal.pone.0006705 (PMC2725320; doi:10.1371/journal.pone.0006705)
Supplement: Table S1 — Analysis of ratios of nodal length to nodal diameter in spinal cord treated with glutamate in vivo. (0.03 MB PDF) [file pone.0006705.s005.pdf]

Table S1. Analysis of ratios of nodal length to nodal diameter in spinal cord treated with glutamate *in vivo.*

Glutamate group (Glut)					Saline group (Ctrl)
Upper 3[e]	Upper 2[d]	Upper [c]	Exposed [b]	Lower [a]	Exposed
		2.87±0.18	3.11±0.31	2.65±0.11	
			3.25±0.15		1.02±0.05
1.27±0.06	2.16±0.09	2.27±0.13	3.29±0.15		0.97±0.03
1.18±0.04	2.33±0.08	2.77±0.10	3.02±0.09		1.04±0.05

[a] 'Lower' means sections 5mm away from the exposed site along the spinal cord toward the tail.
[b] 'Exposed' means sections at the exposed site.
[c] 'Upper' means sections 5mm away from the exposed site along the spinal cord toward the head.
[d] 'Upper 2' means sections 15mm away from the exposed site toward the head.
[e] 'Upper 3' means sections around 30mm away from the exposed site.
Each ratio is the average of around 50 nodes.
Glutamate group includes 4 rats and saline group includes 3 rats.
The statistical data were presented as mean ± s.e.m.
